# Spinal Cord Stimulation as a Potential Therapeutic Modality for Managing Concurrent Chronic Low Back and Abdominal Pain Complicated by Device Migration: A Case Report

**DOI:** 10.1002/ccr3.71784

**Published:** 2026-01-07

**Authors:** Bi Mo, Sandra Sacks, Jerry Markar

**Affiliations:** ^1^ Department of Anesthesiology and Perioperative Medicine, Division of Pain Medicine David Geffen School of Medicine, University of California, Los Angeles Los Angeles California USA; ^2^ Department of Internal Medicine, Division of Hematology‐Oncology University of California, Los Angeles, David Geffen School of Medicine Los Angeles California USA

**Keywords:** chronic abdominal pain, chronic low back pain, complex pain syndromes, device complications, spinal cord stimulation

## Abstract

A 48‐year‐old man with chronic pancreatitis–related chronic abdominal pain (CAP) and concurrent chronic low back pain (LBP) with radiculopathy had inadequate relief from injectable and opioid therapies. A spinal cord stimulation (SCS) trial with dual leads spanning T4–T6 produced significant CAP relief, leading to permanent implantation at T5, after which he reported improvement in both CAP and LBP. Several months later, analgesia abruptly failed due to caudal lead migration to T7 with high impedance at multiple contacts and unsuccessful reprogramming, prompting referral for surgical revision with paddle leads. This case supports SCS for combined CAP/LBP in selected refractory patients while underscoring device‐related complications, such as lead migration, as key limitations requiring close clinical follow‐up.

**Abbreviations**
CAP: chronic abdominal pain
ECAP: evoked compound action potential
FBSS: failed back surgery syndrome
IPG: implantable pulse generator
LBP: low back pain
LLE: left lower extremity
ROM: range of motion
SCS: spinal cord stimulation
T: thoracic
VAS: visual analog scale

## 1 Introduction

Low back pain (LBP) is one of the most common reasons for work absenteeism in the Western hemisphere and a primary contributor to years lived with disability worldwide [1, 2]. Chronic abdominal pain (CAP) is also a leading gastrointestinal complaint prompting outpatient evaluations, affecting up to 25% of the adult population [3]. Both conditions individually can lead to significant functional limitations and are associated with psychiatric disturbances, including depression and anxiety [4, 5].

Common treatment modalities for LBP and CAP include pharmacological therapies, rehabilitation regimens, and injectable therapies such as epidural steroid injections, sensory nerve blocks, and sympathetic plexus neurolysis/ablations [3, 6]. Opioid therapy is often considered when patients' symptoms are refractory to other pharmacological or interventional treatments [1, 7, 8]. However, opioid therapies present challenges, including physiological dependence, development of tolerance over time, and potential for addiction [7, 8]. Additionally, chronic long‐term opioid use may exacerbate underlying symptoms of LBP and CAP due to opioids' pro‐inflammatory nature, effects on gastrointestinal motility, and negative impact on hormonal feedback loops [6, 7, 8, 9]. As a result, there is an imperative need to identify other effective therapies to mitigate symptoms in these patient populations.

Spinal cord stimulation (SCS) is a minimally invasive surgical modality used to treat various chronic pain conditions refractory to conventional therapies [3, 5, 6, 7]. It utilizes electrical signals to interrupt and modulate nociceptive transmission, providing symptom relief [5, 6, 7]. Permanent leads can be implanted surgically or percutaneously and are connected to an implantable pulse generator (IPG) available in rechargeable and non‐rechargeable formats [10]. As an invasive therapy, SCS carries inherent risks, including pain, hematoma, infection, and neurological injury. Device‐related complications such as lead migration and device malfunction are also common reasons for therapy failure, often requiring revisions or device removal [3, 6, 7].

Current indications for SCS include neck and low back pain with or without radicular symptoms secondary to various pathologies, such as cervical or lumbar spine failed back surgery syndrome (FBSS), spondylosis, and radiculopathy [6]. Moreover, applications in treating post‐herpetic neuralgia and diabetic neuropathy in the lower extremities refractory to conservative interventions have demonstrated clinical efficacy [11]. Utilizing SCS for CAP has also been an area of interest, with evidence supporting its clinical efficacy and benefits [3, 5, 6, 7].

SCS therapy, therefore, offers a potential modality to treat two significant pathologies with substantial impacts on individual and societal scales. Nevertheless, no systematic studies have investigated the utilization of SCS to target concurrent LBP and CAP in selected patient populations. We present a case utilizing SCS to treat concurrent LBP and CAP with initial success but complicated by device‐related issues, resulting in loss of therapeutic efficacy.

## 2 Case History/Examination

A 48‐year‐old male presented with concurrent LBP with left lower extremity (LLE) radiculopathy and chronic abdominal pain localized to the epigastric region and radiating to the left upper and lower quadrants, secondary to chronic pancreatitis, gastritis, esophagitis, and splenic venous thrombosis in the setting of a previous history of alcohol and tobacco abuse, requiring prior intensive care unit admission. The patient rated both pain generators from 4 to 8 out of 10 on the visual analog scale (VAS) on average, daily. He described his CAP as sharp and stabbing, while he noted that his LBP and LLE symptoms were primarily aching with intermittent sharp exacerbations and associated dysesthesia. The patient reported postprandial aggravation of CAP along with associated weight loss over time and anemia. The patient also noted activity‐induced aggravation of axial LBP with radicular symptoms into the LLE during range of motion (ROM), lifting, prolonged sitting or standing, and defecating.

The patient reported a history of declining benefits from repeated interventions, including lumbar sympathetic nerve blocks (initially lasting 6 weeks compared to 2–3 weeks at best subsequently) and epidural injections (providing 50% relief for LLE sensory disturbances but no significant relief of low back symptoms) for CAP and LBP respectively. Notably, the patient also reported a history of opioid medication addiction, where he was misusing morphine, hydromorphone, and other opiate derivatives prescribed for his chronic pain, requiring physician‐directed detoxification and discontinuation of therapy. The patient was utilizing acetaminophen 1000 mg four times a day along with gabapentin 600 mg every 8 h for symptom management, and noted inadequate benefits.

Physical examination was significant for significantly reduced ROM of the lumbar spine and pain over bilateral facet regions, along with dysesthesia along the LLE in a nonspecific dermatomal pattern.

## 3 Differential Diagnosis, Investigations and Treatment

**FIGURE 1:**
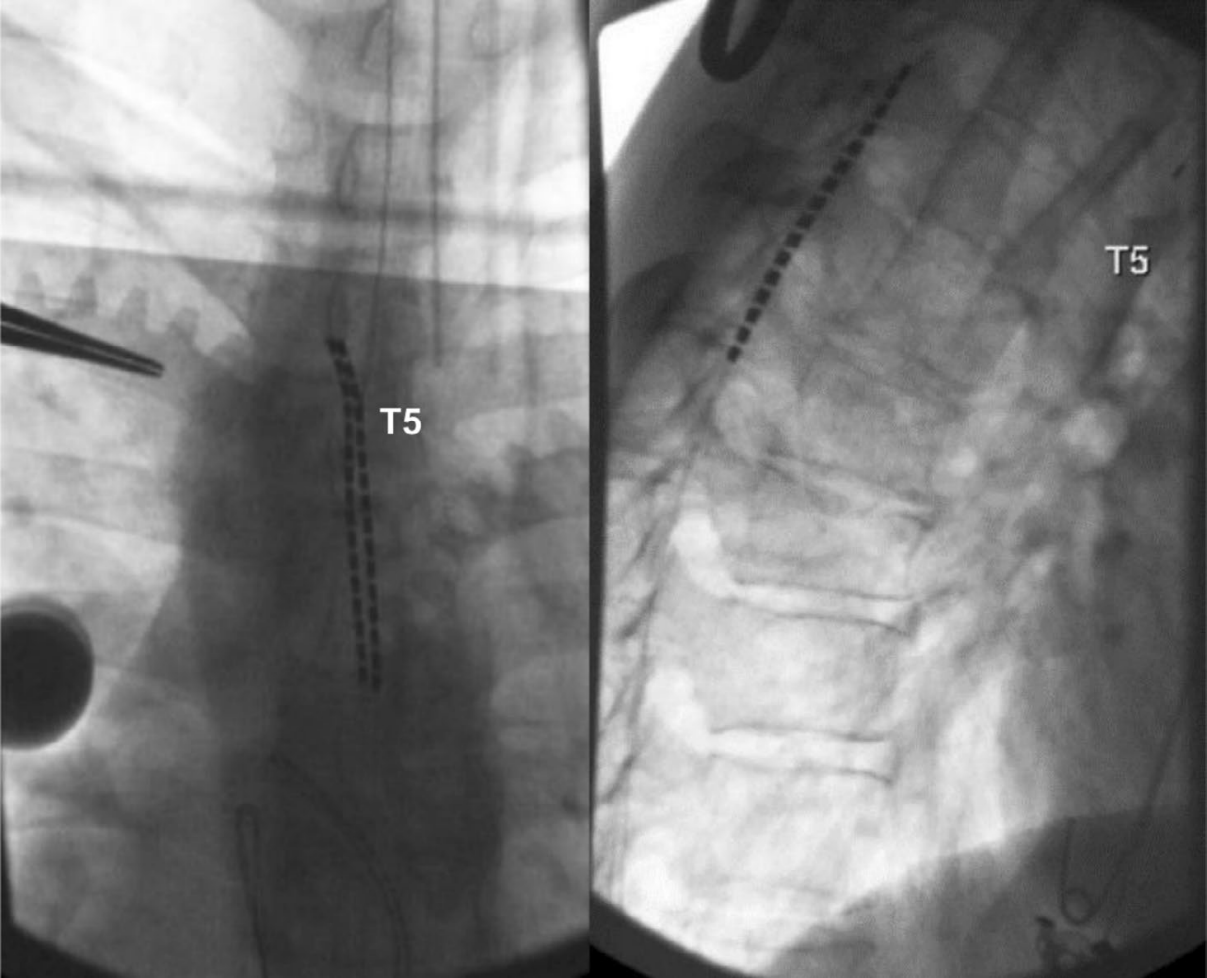
Intraoperative fluoroscopy (anteroposterior and lateral views) indicating position 0 at the superior endplate of T5 and demonstrating anatomical thoracic rotational scoliosis.

The patient underwent an SCS trial with two percutaneous leads placed between the T4 and T6 vertebral body levels and reported 80% relief of CAP, with improved ability to eat and perform physical activities that had previously aggravated his symptoms. There was no documented concurrent improvement in LBP during the 6‐day trial. The patient subsequently underwent permanent SCS implant with percutaneous placement of SCS leads. Both leads were placed with the 0‐position at the superior endplate of T5 (Figure 1). Intraoperative observations were significant for thoracic rotational scoliosis. The intra‐ and perioperative course was uncomplicated, with the patient making an uneventful recovery except for SCS anchor site pain due to lean body habitus.

Post‐permanent implant, it was noted that the patient was receiving predominant mid‐ to right‐sided stimulation compared to left, with a reported 50% reduction of midline and left‐sided CAP symptoms. Concomitantly, the patient also noted more than 50% reduction of his LBP and LLE symptoms with improvement of functional outcomes. Numerous optimization adjustments were made to capture more left‐sided symptoms with moderate success. A decision was made to monitor the patient's response for the next few months prior to further considerations, including potential lead placement revision with intraoperative monitoring given the intraoperative observation of thoracic rotational scoliosis.

**FIGURE 2:**
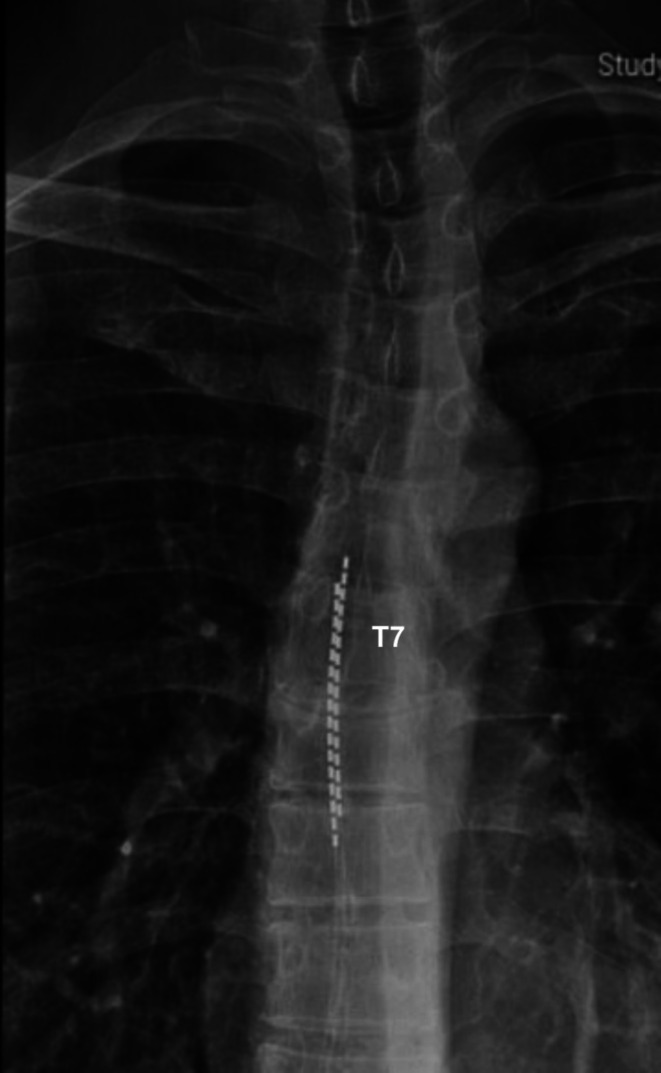
Anteroposterior thoracic plain film revealing caudal migration of the SCS lead, ending at the superior endplate of T7, and demonstrating anatomical rotational scoliosis.

Seven months later, the patient noted an abrupt loss of therapeutic efficacy after moving out of state, with recurrence of severe CAP and concurrent LBP/LLE pain. Outside imaging studies indicated interval caudal migration of both SCS leads two vertebral body levels below, ending at T7 levels, and further displacement to the right (Figure 2). Programming attempted by the SCS manufacturer team also noted high impedance of the left‐sided lead at contact points at positions 5–7 and 13–15. Reprogramming attempts were unsuccessful, and the patient was subsequently referred to a neurosurgeon for surgical revision with paddle leads.

## 4 Conclusion and Results (Outcome and Follow‐Up)

This case demonstrates the therapeutic potential of spinal cord stimulation for concurrent chronic abdominal and low back pain after failure of conservative care, while highlighting pragmatic pitfalls related to device failure, such as lead migration causing abrupt loss of therapeutic efficacy and necessitating surgical revision. From a practice standpoint, several broadly applicable steps emerge: meticulous candidate selection with careful consideration of thoracic target levels when abdominal pain predominates; anticipation of anatomic challenges in the presence of rotational deformity and, when feasible, use of intraoperative neuromonitoring to objectively verify coverage; structured, longitudinal re‐programming; and a low threshold for targeted imaging with early surgical consultation when migration or high‐impedance contacts are suspected. Coordinated, multidisciplinary follow‐up within a multimodal, opioid‐sparing framework supports durability of benefit. Collectively, these observations distill best‐practice insights relevant to both routine and uncommon presentations and translate readily into everyday neuromodulation care.

## 5 Discussion

Etiologies of LBP are commonly degenerative in origin, including lumbar/lumbosacral disc disorders with or without radiculopathy symptoms, spinal canal/foraminal stenosis, with or without concurrent neurogenic claudication in the lower extremities, or persistent pain post‐surgical interventions such as instrumentation, decompression, and total disc arthroplasty, etc. [6]. Nevertheless, the exact mechanism of low back and associated lower extremity symptoms remains elusive, given that imaging phenotypes and clinical findings are not always congruent [12].

CAP has a propensity to affect females more than males [7]. A distinction between abdominal somatic and visceral etiologies is critical to direct effective therapy guidance [3, 5, 7, 8]. Commonly, structural causes include inflammatory bowel disease, such as ulcerative colitis, and chronic pancreatitis, while functional etiologies include gastroparesis, dyspepsia, and irritable bowel syndrome [3, 7]. Post‐surgical abdominal pain after intra‐abdominal interventions such as cholecystectomy, adhesion lysis, and endoscopic retrograde cholangiopancreatography (ERCP) is also a prevalent complaint with significant debilitation of patients' functional outcomes [7].

A concurrent occurrence of both conditions is not only functionally and physically debilitating, affecting basic activities of daily living, but also compounds the significant negative psychological impact on affected individuals [4, 5].

With current data available, SCS is more commonly applied in the treatment of chronic LBP and lower extremity radicular pain than CAP, with numerous studies demonstrating clinical benefits [3, 5, 6, 7, 13]. Multiple studies have shown that SCS can meaningfully reduce CAP pain scores and associated symptoms (including nausea and emesis) and can also decrease opioid requirements for symptom control [3, 7]. Notably, Kapural et al. [3, 7] demonstrated in their retrospective and prospective studies promising outcomes in CAP patient cohorts with long‐term efficacy post‐SCS therapy, with clinically significant reduction of symptoms and associated requirement for opioid therapies. Especially with the advent of evoked compound action potential (ECAP)‐controlled, closed‐loop systems with automated dynamic feedback strength adjustments, further systematic studies evaluating this latest development in central nervous system neuromodulation would shed further light on the therapeutic value of SCS therapy in challenging clinical settings [14]. Nevertheless, consistent with the limitations demonstrated in our case report, device‐related complications present significant challenges to the continuation and durability of SCS therapy [3, 5, 7].

Given these risks, SCS should be managed as a dynamic therapy requiring ongoing assessment and iterative optimization [13]. Notably, any abrupt loss of efficacy of therapy should raise concerns about device‐related complications, including lead displacement, migration, breakage, and IPG malfunction [6, 15]. Targeted clinical studies, including plain film X‐rays to evaluate lead position and lead integrity, should be obtained to evaluate and investigate potential underlying causes.

Chronic pain is also often associated with significant psychosocial elements, most commonly depression and anxiety, which can exacerbate underlying pain syndromes [4]. These factors present a barrier to functional and psychological recovery, and adversely impact patients' perception of pain and response to SCS [11]. Accordingly, this correlation underscores the critical importance of a comprehensive approach that includes psychological evaluation and support not only perioperatively but also on an ongoing basis to optimize subsequent therapeutic response to neuromodulation therapies.

## Author Contributions

**Bi Mo:** conceptualization, data curation, formal analysis, visualization, writing – original draft, writing – review and editing. **Sandra Sacks:** data curation, formal analysis, writing – original draft, writing – review and editing. **Jerry Markar:** data curation, formal analysis, writing – original draft, writing – review and editing.

## Funding

The authors have nothing to report.

## Ethics Statement

Per UCLA IRB policy, a case report without identifying information does not require IRB approval.

## Consent

Written informed consent was obtained from the patient for the publication of this case report and any accompanying images. A copy of the written consent is available for review upon request by the editor(s).

## Conflicts of Interest

The authors declare no conflicts of interest.

## Data Availability

Data sharing not applicable to this article as no datasets were generated or analysed during the current study.
